# Stereotactic Radiotherapy for the Management of Refractory Ventricular Tachycardia: Promise and Future Directions

**DOI:** 10.3389/fcvm.2020.00108

**Published:** 2020-06-25

**Authors:** Raphael Jumeau, Mahmut Ozsahin, Juerg Schwitter, Olgun Elicin, Tobias Reichlin, Laurent Roten, Nicolaus Andratschke, Michael Mayinger, Ardan M. Saguner, Jan Steffel, Oliver Blanck, Marie-Catherine Vozenin, Raphael Moeckli, Michele Zeverino, Véronique Vallet, Claudia Herrera-Siklody, Patrizio Pascale, Jean Bourhis, Etienne Pruvot

**Affiliations:** ^1^Department of Radiation Oncology, Lausanne University Hospital and University of Lausanne, Lausanne, Switzerland; ^2^Multidisciplinary Cancer Care Service, Radiation Oncology Unit, Riviera-Chablais Hospital, Rennaz, Switzerland; ^3^Heart and Vessel Department, Cardiac MR Center, Lausanne University Hospital and University of Lausanne, Lausanne, Switzerland; ^4^Department of Radiation Oncology, Inselspital, Bern University Hospital and University of Bern, Bern, Switzerland; ^5^Department of Cardiology, Inselspital, Bern University Hospital and University of Bern, Bern, Switzerland; ^6^Department of Radiation Oncology, University Hospital Zurich, Zürich, Switzerland; ^7^Department of Cardiology, University Heart Center Zurich, Zürich, Switzerland; ^8^Department of Radiation Oncology and Department of Internal Medicine III, Cardiology, Section for Electrophysiology, University Medical Center Schleswig-Holstein, Kiel, Germany; ^9^Radio-Oncology Research Laboratory, Lausanne University Hospital and University of Lausanne, Lausanne, Switzerland; ^10^Institute of Radiation Physics, Lausanne University Hospital and University of Lausanne, Lausanne, Switzerland; ^11^Heart and Vessel Department, Service of Cardiology, Lausanne University Hospital and University of Lausanne, Lausanne, Switzerland

**Keywords:** ventricular tachycardia, radiotherapy, stereotactic radiotherapy, ablation, noninvasive

## Abstract

Ventricular tachycardia (VT) caused by myocardial scaring bears a significant risk of mortality and morbidity. Antiarrhythmic drug therapy (AAD) and catheter ablation remain the cornerstone of VT management, but both treatments have limited efficacy and potential adverse effects. Stereotactic body radiotherapy (SBRT) is routinely used in oncology to treat non-invasively solid tumors with high precision and efficacy. Recently, this technology has been evaluated for the treatment of VT. This review presents the basic underlying principles, proof of concept, and main results of trials and case series that used SBRT for the treatment of VT refractory to AAD and catheter ablation.

## Introduction

Ventricular tachycardia (VT), an important cause of mortality and morbidity (1), commonly occurs in the context of structural heart diseases (e.g., post-myocardial infarction). Recent progress in cardiac imaging and electroanatomic mapping (EAM) techniques have prompted the use of catheter ablation (CA) for VT substrates delineation and ablation (2, 3). Antiarrhythmic drug therapy (AAD) and CA are the cornerstone of VT management, but both treatments have limited efficacy and potential adverse effects (4, 5). Additionally, despite significant progress in CA efficacy, the recurrence rate after a first VT ablation is about 50% (6), exposing patients to multiple CA procedures (7) and implantable cardioverter-defibrillator (ICD) shocks (8).

Stereotactic radiotherapy (RT), routinely used in the realm of oncology to non-invasively treat solid tumors with high precision and efficacy, appears as a new tool in VT management (9). We sought to review the current literature in order to summarize data and perspectives of this innovative technique in the management of VT.

## Ventricular Tachycardia: Pathophysiology, Current Management, and Limitations

**Figure 1:**
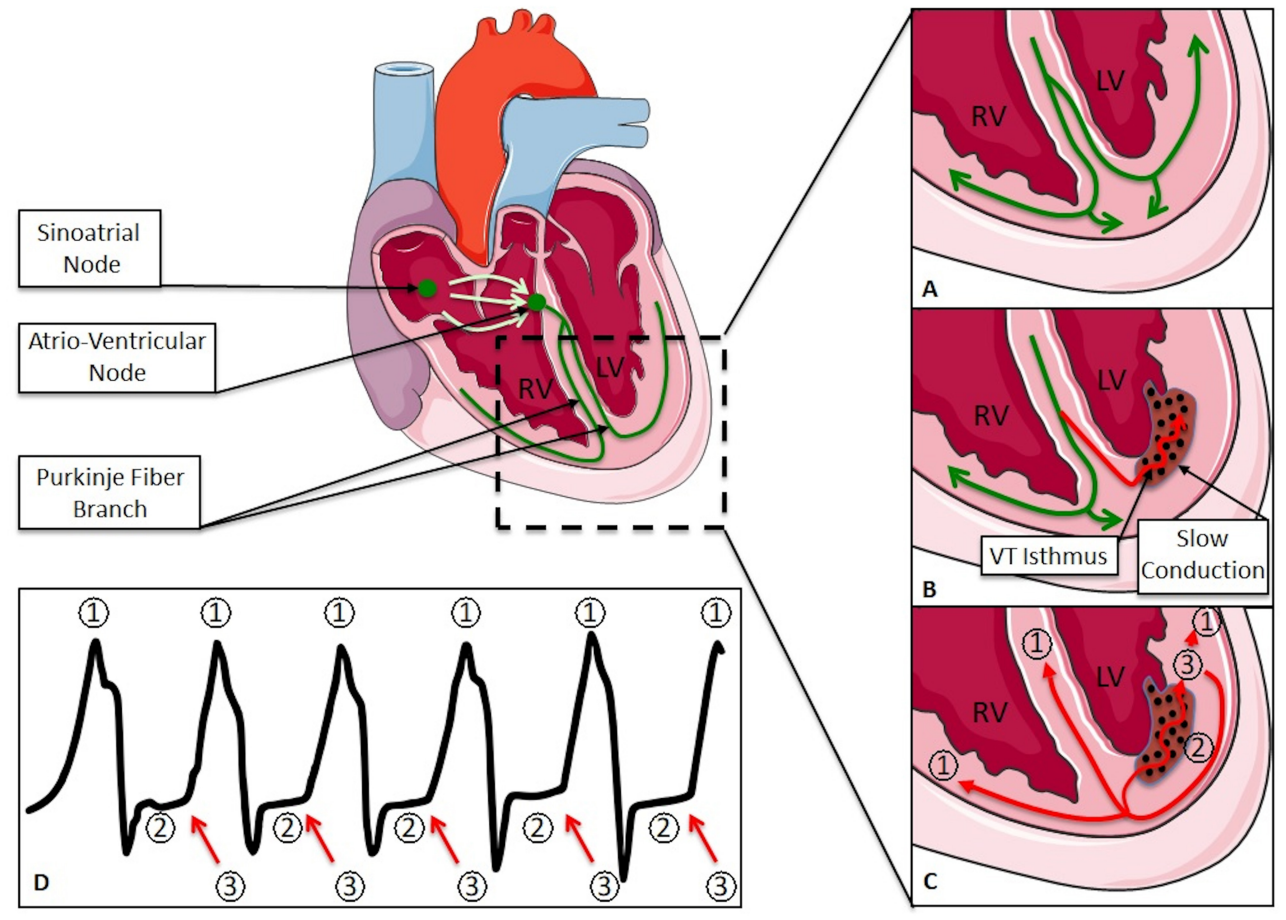
**(A)** Normal progression of the activation wavefront; **(B)** slow conduction after myocardial infarction; **(C)** re-entrant circuit; **(D)** ECG of a ventricular tachycardia: (1) depolarizing wavefront; (2) slow conduction; (3) exit site. RV, Right Ventricule; LV, Left Ventricule.

VT may be idiopathic or caused by an underlying myocardial substrate that initiates the arrhythmia (e.g., ventricular premature contraction, VPC) and/or maintains the re-entrant circuit. These abnormal anatomical structures are often the result of pathologic changes like post-myocardial infarction scars or post-inflammatory scars. Re-entry is the most common mechanism of VT in this setting (Figure 1). Re-entrant circuits utilize surviving myocytes within or at the border zone of scars resulting in an isthmus (Figure 1) of slow electrical conducting fibers of variable refractoriness (10). Connection of the isthmus to the healthy myocardium determines the site of initial ventricular activation (i.e., exit site, Figure 1) that drives the ECG appearance of the VT. Cardiac inflammatory diseases and post-inflammatory remodeling may also lead to scar and VT susceptibility (11).

Developed in the late 70's, ICDs have revolutionized the approach to the prevention of sudden cardiac death after myocardial infarction (12). ICDs are nowadays commonly used to detect and promptly treat malignant ventricular arrhythmias (13). The benefit of ICDs derives from the ability to interrupt re-entrant VT by anti-tachycardia pacing or shocks. Although these therapies may be life saving, ICD shocks may be traumatic and decrease quality of life (14). Importantly, ICDs are very effective in treating VT episodes but lack any preventive effect.

AAD to supress VT have poor efficacy and multiple side effects that limited their use (4). Interventional therapies, such as CA that disrupts or alters the VT substrate have become the standard of care (6). Most CA techniques currently utilize radiofrequency energy to heat cardiac tissue leading to tissue necrosis, and consequently to the disruption of the VT substrate and circuit. Two approaches have been used over the last decade. The first targets the isthmus of the VT in order to interrupt the re-entrant circuit. The second one, named *substrate modification*, targets in sinus rhythm any surviving myocardial fibers within or at the border zone of the scar that could serve as potential VT isthmuses because of their slow conducting properties. Fractionated potentials and late activated ventricular potentials are typical examples. Cryoablation, which utilizes a freezant to destroy the VT substrate, has gained increased utilization recently (15). Although CA techniques can be performed for most tachyarrhythmias, their use are also limited by incomplete efficacy, unfavorable side effects, and procedural risks (16). The recurrence rate after CA is about 50% at 2 years for ischemic VT, independently of the chosen approach (substrate modification vs. VT isthmus ablation), with even higher recurrence rates for intramural (e.g., in the interventricular septum) and non-ischemic VTs (17).

For patients with VT refractory to standard treatments, new techniques are described in the literature such as surgical epicardial ablation, video-assisted thoracoscopic cardiac sympathetic denervation (CSD), or intracoronary ethanol infusion (18) and other means under extensive study such as bipolar radiofrequency ablation, half-normal saline irrigation, or needle electrodes (19). Despite limited series, CSD might be an interesting option in patients with sustained VT who have failed both antiarrhythmic medication and catheter ablation for refractory VT (20, 21). However, the antiarrhythmic properties of CSD on VTs arising from structural heart disease need to be deciphered and documented on larger series. To date, there are no data on the combination of alternative techniques with SBRT. The development of non-invasive complementary therapies such as SBRT appears to be an alternative for the most fragile patients and/or those with slow VT.

## From Conventional Radiotherapy to Stereotactic Body Radiotherapy

**Figure 2:**
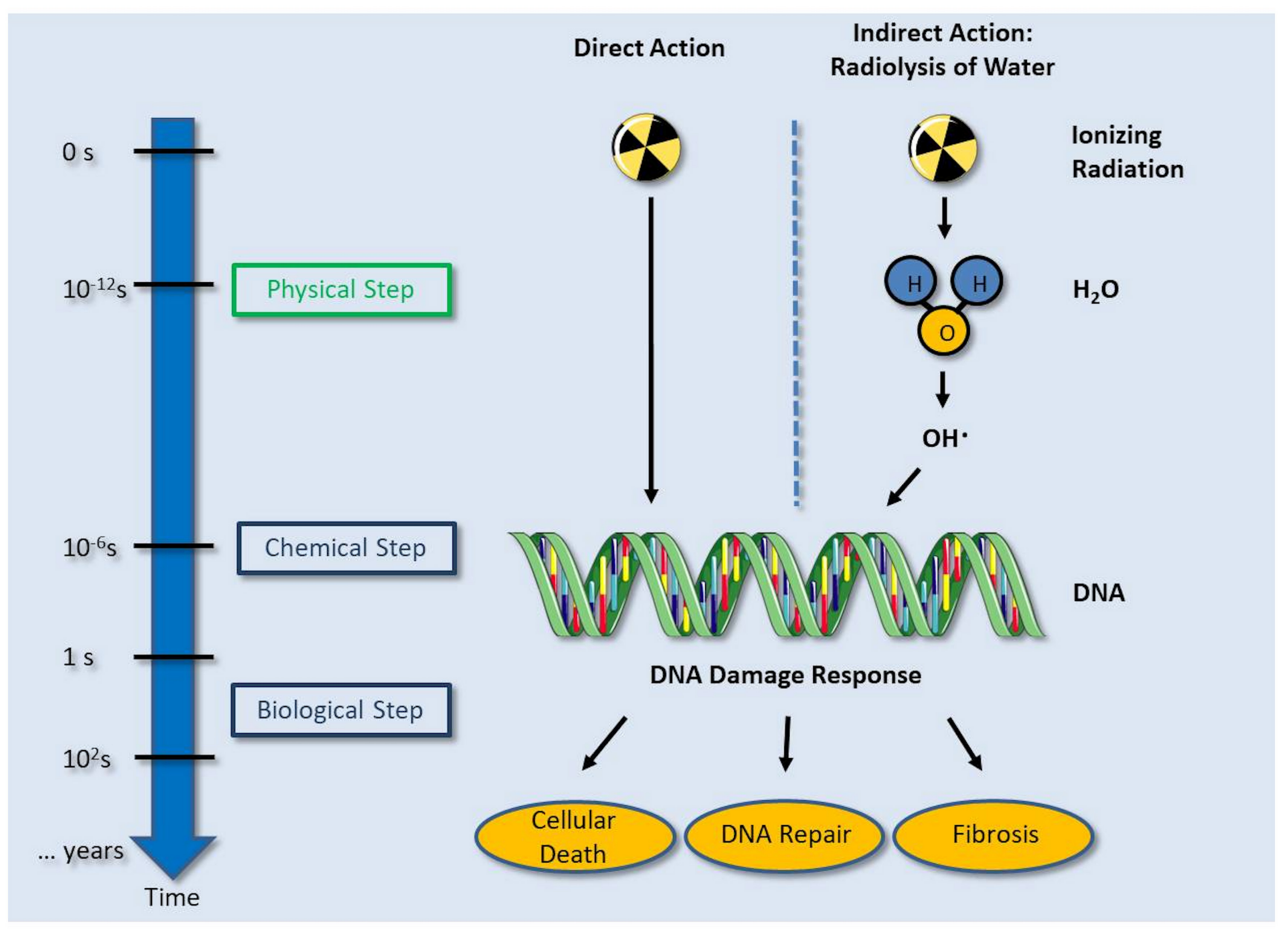
Biological effect of X-ray (ionizing radiation) on human tissue.

**Figure 3:**
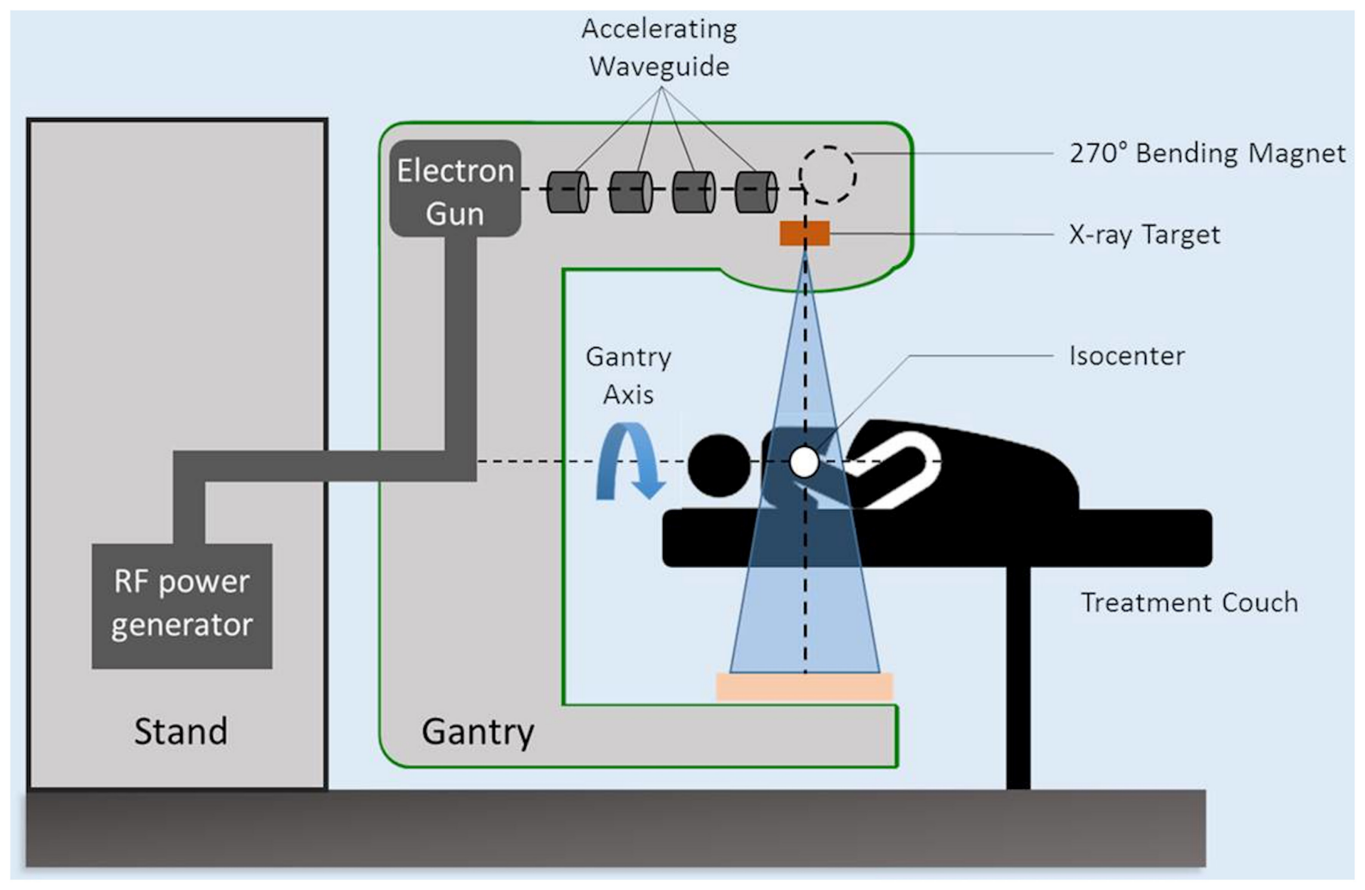
Schematic diagram of a typical linear accelerator (linac).

**Figure 4:**
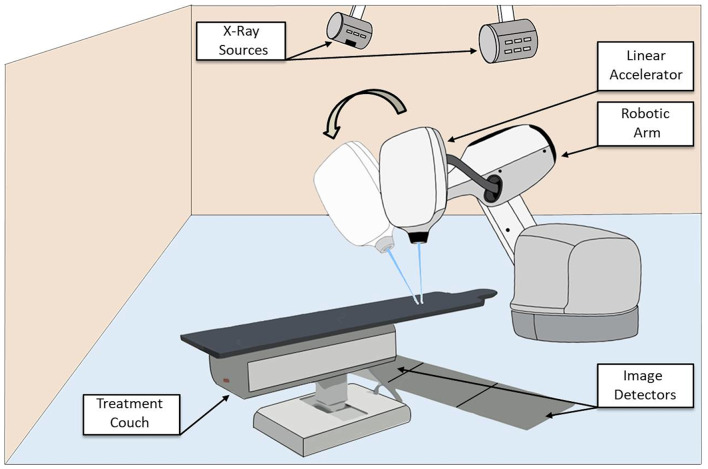
Schematic diagram of the CyberKnife® system.

RT utilizes high energy X-rays from linear accelerators (linac) to destroy the targeted tissue, most commonly cancer (Figures 2–4). Historically, conventional RT as exclusive treatment of cancer consisted of multiple daily fractions of irradiation over 4–8 weeks at a dose of 1.8–2 Gy per fraction, leading to high cure rates of various cancers (e.g., prostate, lung, head, and neck). The therapeutic effect of RT is influenced by the dose per fraction and the cumulative total radiation dose, the number of fractions, and the total delivery time. However, the impact on healthy tissues is usually the limiting factor in the total dose delivered to a target tissue (usually the tumor). During the last decades, several technical innovations were introduced in radiation oncology. Modern RT techniques, thanks to on-board imaging systems that check the position of the target and organs at risk (OARs) before treatment, allowed to increase dose delivery to the target volume, while reducing the dose to OARs, and consequently acute and late toxicities (22, 23).

In parallel, a change in the fractionated RT treatment paradigm arose with the advent of brain radiosurgery (24). Radiosurgery usually consists of 1–5 fractions of very high-dose RT (>6 Gy/fraction) delivered with stereotactic accuracy (25). The application of radiosurgical principles outside the brain is termed stereotactic ablative radiotherapy (SABR) stereotactic body radiotherapy (SBRT). Compared to conventional RT, SBRT ablates the targeted tissue with a minimum of fractions. This is supported by radiobiologic studies showing that tumor response is improved with larger doses of radiation delivered in fewer fractions (26, 27). Consequently, due to the high doses also delivered to nearby OARs, SBRT is adapted to small volume targets.

To achieve the necessary high level of accuracy for SBRT dedicated RT devices have been specifically developed for stereotactic treatments at various cancer sites (28). The TrueBeam® system (Varian Medical Systems, Palo Alto, CA), based on a linac, provides image-guided SBRT thank to the on-board cone beam CT and the 6-dimensional couch. Alternatively, the CyberKnife® system (Accuray, Sunnyvale, CA, USA) is an image-guided device dedicated to radiosurgery and SBRT (Figure 4) (29). This device is mounted on a robotic arm to deliver radiation to a tumor from different trajectories, while minimizing dosage to adjacent normal tissue. The CyberKnife® is also able to track tumors directly or alternatively fiducial markers placed in the vicinity of the tumor to deliver highly accurate treatments in order to minimize the dose to surrounding tissues and OARs (30). Recently, RT devices combining linac and medical resonance imaging (MRI) system have been developed fort SBRT treatments (31).

Radiosurgery for non-oncologic diseases is most commonly performed for neurologic disorders or benign central nervous system tumors (32). The most extensively developed data for radiosurgical treatments have pertained to treatment of vestibular schwannoma (33), meningioma of the skull base (34) or seizure (35). Radiosurgery has the advantage of being delivered on an outpatient basis, and has become an excellent alternative to invasive neurosurgery for organ preservation in selected patients.

## Heart and Radiotherapy: A Complex Combination

Providing RT for a non-oncologic disease, such as within the heart, may appear paradoxical given the well-known long-term side effects of RT on cardiac tissue. It is important to note that age and pre-existing heart conditions are well-established risk factors for cardiotoxicity of any kind, including radiation-induced cardiac toxicity. Therefore, patients undergoing SBRT for refractory VT are likely to be a risk category (36). Radiation induced cardiac toxicity (RICT) is a late complication of RT, with increasing risk over years after treatment (37, 38). The use of RT contributed to significant survival improvements for patients with breast, lung, esophageal, lymphoma, and thymic cancers. These successes resulted in large cohorts of cancer survivors, who were subject to late or very late complications from RT (39–43). Depending on indication, dose and RT techniques, any subparts of the heart can be damaged such as the pericardium, myocardium, heart valves, coronary arteries, capillaries, and conduction system.

The pathophysiological pathway that leads to RICT suggests that radiation causes both microvascular and macrovascular damages (44). The microvascular injury is characterized by a decrease in capillary density, causing myocardial ischemia and fibrosis. Fibrosis can lead to several consequences: valves dysfunction, pericardial fibrosis (45), and/or effusion, arrhythmia (46, 47), and loss of cardiac compliance leading to diastolic dysfunction (48). Macrovascular injury may manifest as accelerated coronary atherosclerosis (49).

With the emergence of modern RT techniques, heart structures can be much better spared during treatment. Additionally, once RICT was recognized, treatment techniques were modified to minimize cardiac irradiation such as the development of deep inspiration breath-hold techniques during RT for left breast cancer (50). Although there is no minimal safe radiation dose, more recent data suggest a decrease of the dose received by the heart with modern RT techniques, reducing most probably RICT incidence (51).

Currently, heart dose constraints during a thoracic RT are based on the dose received by the whole cardiac volume. To date, there are limited data on the correlation between the dose received by cardiac substructures (valves, coronary arteries, etc.) and potential side effects, and no specific dose constraints validated for each of these substructures. Hahn et al. (52) observed in patients who received mediastinal RT for Hodgkin lymphoma that the risk of late ischemic cardiac events is correlated with the dose received by the coronary arteries such as: volume of left anterior descending artery receiving 5 Gy and volume of left circumflex artery receiving 20 Gy in conventional fractionation (1.8–2 Gy per fraction).

**Figure 5:**
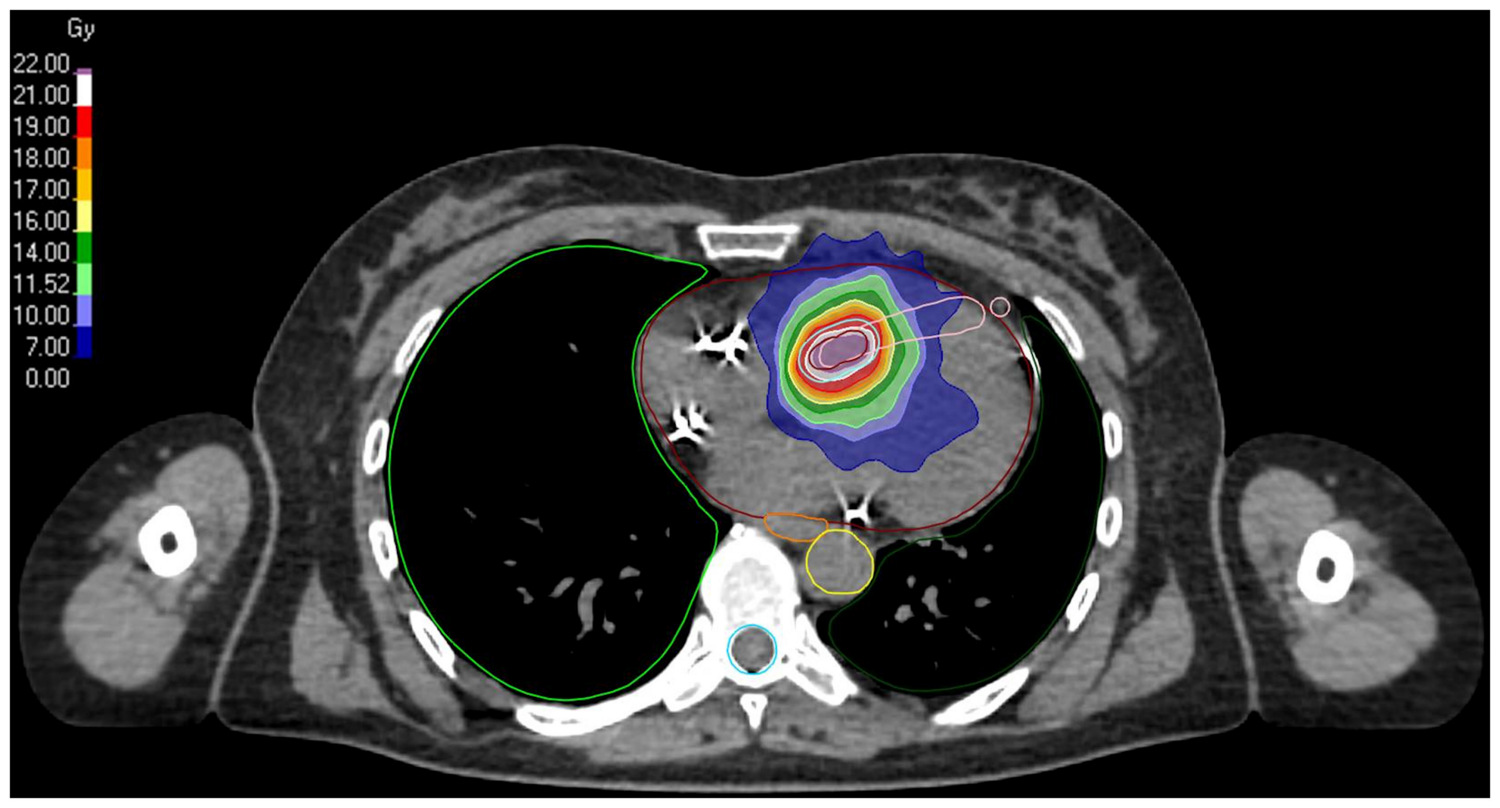
Stereotactic body radiotherapy (SBRT) plan for a ventricular tachycardia (VT) arising from the interventricular septum. The planning target volume is in light blue, the heart is in dark red, the right lung is in green, the left lung is in dark green, the esophagus is in orange, the spinal canal is in blue, the interventricular septum is in pink and the aorta is in yellow.

SBRT appears as a more attractive modality to irradiate the heart than conventional RT. Thanks to the highly accurate targeting provided by detailed electroanatomical mapping of the arrhythmia, a better dose fall-off in all directions compared to conventional RT and the possibility to spare cardiac substructures, acute, and long-term toxicities may be minimized (Figure 5) (53). An additional difficulty for cardiac SBRT, compared to the field of neurology, is the presence of a moving target. Indeed, the heart is submitted to its own internal movements and also to breathing. These movements can be taken into account by using RT devices equipped with a tracking system or by the addition of margins corresponding to internal movements (Internal Target Volume, ITV) for linac-based systems.

## From Pre-Clinical Data to First Patients

In preclinical studies in healthy animals, the proof-of-principle was demonstrated by irradiating heart tissue to create fibrosis similar to CA. Sharma et al. (54) suggested that a single dose of at least 25 Gy on cavo-tricuspid isthmus, AV node, or pulmonary veins is needed to create a lesion that alters electrophysiological properties. The timeline showed an electrophysiologic effect consistently over 90 days. In 2011, Maguire et al. (55) showed that a single fraction of 25–35 Gy on mini pig pulmonary veins was able to create transmural fibrosis that resulted in electrical isolation 6 months after irradiation. Similarly, Blanck et al. (56) showed that doses >32.5 Gy in the healthy pig heart can induce transmural scarring of cardiac tissue (electrophysiology study 6 months after irradiation). Yet, the underlying electrophysiologic effects of the fibroting and sub-fibroting doses (e.g., <30 Gy) remained unknown beyond healthy animal models. Some additional data could be obtained from pre-clinical studies with carbon ions or protons whose radiobiology might be slightly different as compared to photons (57). Of note, pre-clinical data about scar development at the ventricular level (temporal onset and dose-response relationship) after high dose irradiation are very limited since most studies reported data about atrial tissue (pulmonary veins or cavo-tricuspid isthmus) or AV node irradiation.

To date, clinical outcome of SBRT for cardiac arrhythmias is limited to only small prospective and retrospective case series (Table 1) and case reports. Loo et al. (58) reported the first patient treated with SBRT for AAD-refractory VT after myocardial infarction. The VT substrate was made of surviving myocardial fibers within the scar according to the PET-CT. The treatment was delivered with the CyberKnife® with a temporary pacing wire placed at the right ventricular apex to ensure accuracy (i.e., function as fiducial marker). A similar report by Cvek et al. (59) described the first procedure using the CyberKnife® system in Europe in a patient suffering from a dilated cardiomyopathy. The VT substrate location was based on an electrophysiological (EP) study using an EAM system (CARTO3, Biosense Webster, Irvine, CA, USA). Both treatments were successful and delivered 25 Gy in a single fraction, in imitation of the lowest dose with any effect from the preclinical studies.

**Table 1:** Largest case series of cardiac SBRT for refractory VT.

	**Washington University, USA**	**Ostrava University, Czech Republic**	**Emory University, USA**	**Lausanne University Hospital, Switzerland**
Number of patients	19	10	10	10
Age	66 (49–81)	66 (61–78)	61 (51–78)	66 (47–75)
Cardiopathy: I/NI/INFL	11/6/2	7/2/0	4/6/NA	3/4/2
LVEF (%)	25 (15–58)	26.5 ± 3.2	NA	37 ± 14
RT device	Linac	CyberKnife System	Linac	CyberKnife System
PTV margin (mm)	5	0	1–5	0–3
PTV (ml)	98.9 (60.9–298.8)	22 (14.2–29.6)	81.4 (29–238)	23 (14–35)
Dose (Gy)	25	25	25	22 (20–25)
Toxicity	• 1 case with heart failure exacerbation • 1 case of radiation pericarditis • 2 cases of radiation pneumonitis	• 4 cases with nausea • 1 case of possible mitral regurgitation worsening at 17 months	• 2 cases of radiation pneumonitis	• 1 case of nausea • 1 case of broken rib
VT burden reduction	94% at 13 months	87.6% at 28 months	69% at 5.8 months	99.4% at 4 months

*VT, Ventricular Tachycardia; Linac, Linear accelerator; PTV, Planning Target Volume; I, Ischemic; NI, Non-Ischemic; INFL, Inflammatory; NA, Not available*.

The first systematically investigated patients cohort was reported by Cuculich et al. (60) in 2017. In a series of five patients, they reported a strong VT burden reduction of 99.9% after a 6-week blanking period. To determine the location of the VT substrate they used a completely non-invasive mapping method combining a high density surface electrocardiographic imaging technique (ECG-vest with 252 electrodes) that targeted the exit site of the VT and the surrounding ischemic substrate (i.e., infarction and its border zone), merged with a chest CT. All patients received 25 Gy in a single fraction (mean ablation volume of 49 cc) delivered using a linac dedicated for SBRT.

Cuculich et al. then initiated a prospective phase I/II study: ENCORE-VT (NCT02919618) aimed to primarily demonstrate short-term safety and secondarily preliminary efficacy of SBRT for patients with life-threatening, AAD-refractory VT. Mapping of the VT substrate, SBRT delivery, and dose prescription (25 Gy) were similar as for their first patients (60). The main efficacy endpoint was any reduction in VT episodes or any reduction in premature ventricular contractions burden during the 6 months before and after treatment (with a 6-week blanking period after SBRT). First results of this trial with a median follow-up of 13 months have been recently published by Robinson et al. (61). A total of 19 patients were enrolled, with remarkable efficacy in VT burden reduction. The median VT substrate volume (i.e., Gross Target Volume, GTV) was 25.4 cc, the median ITV volume (taking movements into account) was 31 cc and the median final ablation volume (i.e., Planning Target Volume, PTV) was 98.9 cc. The median number of VT episodes decreased from 119 (range, 4–292) in the 6 months before to 3 (range, 0–31) in the 6 months after SBRT. ICD shocks were also significantly reduced from a median of 4 (range, 0–30) to 0 (range, 0–7). Regarding toxicity, 2 patients (10.5%) experienced grade 3 treatment-related adverse events (heart failure and pericarditis) within 90 days of SBRT delivery. In addition, six patients (28%) had treatment-related pericardial effusions including one grade 3 and one grade 4; 11% of patients had pneumonitis that resolved with steroids. The authors recently presented longer-term results showing a persistent effect of SBRT 2 years after treatment in most patients. Additionally, serious toxicity was low, but may occur after 2 years: two grade 3 pericardial effusions and one grade 4 gastro-pericardial fistula (62).

Furthermore, Neuwirth et al. (63) published a larger retrospective case series on SBRT in VT patients refractory to CA with longer term follow-up. Mapping of the VT substrate was based on an electrophysiological (EP) study using an EAM system (CARTO3, Biosense Webster, Israel) as for their first patient (59) and SBRT was delivered using the CyberKnife® system with a single fraction of 25 Gy (mean PTV of 22.2 cc). Results of this series have been recently published at a median follow-up of 28 months. Ten patients were treated, with a VT burden reduction of 87.5% compared to baseline, however, after a 3-month blanking period, VT recurred in 8/10 patients. Finally, they report one possible treatment-related toxicity (mild nausea) and a possible grade 3 late treatment related toxicity (progression of mitral regurgitation 17 months after SBRT). To further study the long-term safety and efficacy of SBRT for TV, the authors have initiated a multicenter prospective study (NCT03819504) (64).

A case series has been published by Lloyd et al. (65) showing the interest of SBRT for refractory VT in advanced heart failure patients. A total of 10 patients with VT refractory to standard treatments were included and received a single fraction of 25 Gy using a linac system dedicated for SBRT with a mean PTV of 81.4 cc. Among eight patients with available ICD data, the total reduction in seconds of detected VT was 69% and the reduction in total ICD shocks after SBRT was 68%. It should be noticed that in this study no blanking-period was considered. They concluded that SBRT for refractory VT was feasible and modestly effective at reducing VT burden in advanced heart failure patients. Interestingly, three patients had post-SBRT histology since they received heart transplant after treatment. Microscopic analyses of the treated regions showed oedema and vacuolization of endothelial cells with mild fibrosis. Electron microscopy of one sample revealed disruption of intercalated disc/gap junction area.

Our group described the first immediate and durable response to cardiac SBRT in an intensive care patient suffering from an electrical storm (ES) due to incessant VT unresponsive to CA and AADs (66). An EP study performed with an EAM was used to delineate the VT substrate location. The right ventricular ICD lead served as a fiducial marker for tracking with the CyberKnife® system. A total dose of 25 Gy in a single fraction was delivered, while the patient was intubated and sedated in the treatment room. Surprisingly, the SBRT rapidly controlled the ES, allowing the extubation of the patient 3 days after the procedure without any recurrence thereafter. To date, our group has treated 10 patients with VT refractory to AAD and CA (67). Eight patients were elective, while the other two (one corresponding to the previously cited patient) were hospitalized in the intensive care unit. All patients had an EAM prior SBRT to define the VT substrate. The mean dose of 22 Gy (range, 20–25) was delivered to the VT substrate (mean PTV of 23 cc) using the CyberKnife® system. At a median follow-up of 6 months (range, 1–14), the elective patients did not experience any sustained VT recurrence or ICD shock. Importantly, no detectable severe adverse events related to SBRT occurred (Table 1).

Recently, another prospective study published by Gianni et al. (68) conducted on five patients mitigates these results. Indeed, the 1-year follow-up reports a recurrence of ventricular arrhythmias in all patients, despite an initial reduction observed in the first 6 months post-ablation. Interestingly, three patients had a redo procedure, which showed surviving bundles of cardiomyocytes within the putative PTV.

Although clinical outcomes of 20–25 Gy single-dose SBRT for refractory VT remain limited to small series and few case reports, results of this new technique are very promising. A longer follow-up on larger cohorts is warranted to assess the efficacy and safety of this technique.

## Perspectives

The efficacy of cardiac SBRT has been attributed to radiation-induced fibrosis (Figure 2) that creates conduction blocks within the heart. However, the reduction of VT episodes within a couple of days after a dose of 25 Gy in the rescue procedure reported by Jumeau et al. (66) suggests that the mechanisms of action may not only be attributed to radiation-induced fibrosis. Similarly, in the series by Cuculich et al. (60), all patients presented a strong reduction in VT episodes within the first month after SBRT, which may not only be attributed to late radiation-induced fibrosis which is usually observed months later. These data support an immediate benefit of SBRT on the VT substrate by other mechanisms, which is consistent with preclinical data showing that single fraction doses above 30 Gy are necessary to create dense, and transmural fibrosis in the heart (56). *In vivo* data by Fajardo and Stewart (69) in animal models showed the presence of inflammatory cells in heart tissue within hours after heart irradiation that could explain this early response. Another interesting experimental finding is that RT might be antiarrhythmic by restoring localization Connexin 43 at intercalated disks both in normal and post-mocardial infarction tissue, which was associated with improved conduction properties and reduced repolarization dispersion (70). There is therefore a need to better understand the pathophysiological mechanisms of SBRT on heart tissue and more specifically on the VT substrate.

While clearly promising, these first steps represent only the beginning of a journey to introduce SBRT into the treatment of cardiac arrhythmias. It is essential to make the technique as safe as possible to avoid toxicities by minimizing unnecessary irradiation of the heart and surrounding tissues. The two main parameters that influence this “unnecessary” irradiation are the ablation volume (i.e., VT substrate) and the prescribed dose. Therefore, there is a need to determine the minimal radiation dose level capable to maintain the efficacy of SBRT for the treatment of cardiac arrhythmias. Importantly, the dose of 25 Gy used so far in cardiac SBRT for VT ablation is relatively high compared to other benign diseases: 18–20 Gy in a single fraction for an arterio-venous malformation (71) or 18 Gy for seizure (35). By reducing the target dose to a minimal effective level, the dose to the surrounding tissue will be reduced, which will lower the probability of long-term complications.

It is also important to keep in mind that the VT substrate is related to the underlying heart disease, e.g., in ischemic VT the substrate is usually delineated, confluent, and limited to the distribution area of a coronary epicardial vessel. In non-ischemic dilated cardiomyopathy, arrhythmogenic cardiomyopathy, or myocarditis/sarcoidosis, the substrate is usually scattered and involves a larger area of the heart. Additionally, the ablation volume is not only related to the VT substrate volume, but also to the SBRT technique. In fact, depending on the SBRT device or on the tracking method, additional geometric safety margins for uncertainty are needed that increase the final ablation volume (i.e., planning target volume, PTV). This has been well-observed in the series of Cuculich and Robinson et al. (60, 61) where the non-invasive mapping of the VT exit (and not of the VT isthmus) and of the ischemic substrate combined with a free-breathing linac based treatment resulted in high PTV dimensions. This has been investigated by Knutson et al. (72) in a dosimetric analysis from ENCORE-VT trial that showed a decrease of PTV between the first and the last treated patient (i.e., learning curve). Hence, wider dose spreading (Figure 1) to OARs could promote pneumonitis and pericarditis.

Recent advances in medical imaging and RT have improved target definition and tracking accuracy. New image-guided RT techniques are currently emerging, including MRI-linac that can combine the possibility of continuous non-ionizing imaging with direct target tracking (73). In the future there is probably a lot to expect from protons and carbon ions as many pre-clinical studies are based on these particle beams (74); a first VT patient has been recently treated using protons (75).

Finally, as with any novel technological advance, radiation therapy for the treatment of cardiac arrhythmias will ultimately have to be tested in well-controlled clinical trials in order to adequately assess the benefits and risks associated with this promising approach. A multi-center, multi-platform clinical feasibility trial on the initial safety profile of radiosurgery for ventricular tachycardia (RAVENTA, NCT03867747) is now recruiting in Germany. To date, several studies have also begun or are in preparation in Milan, Italy (NCT04066517); Calgary, Canada (NCT04065802) or Amsterdam, The Netherlands (NL7510). Before results of these trials with clearly defined protocols become available, this therapy needs to be limited to large centers with collaborative network knowledge in order to optimize patient benefit and safety.

## Conclusions

Cardiac SBRT only recently emerged as a promising treatment option for the management of refractory VT. It appears to be an effective and non-invasive option. Given the recentness of this technology and the scarcity of prospective clinical data with limited long-term follow-up, further research and clinical experience are warranted within prospective clinical trials.

## Author Contributions

RJ and EP: conception and design. All authors: administrative support and final approval. RJ, MO, EP, and JB: collection and assembly of data. RJ, OB, and EP: data analysis and interpretation.

## Conflict of Interest

OE reports personal fees from Merck Serono, personal fees from MSD Global, personal fees from AstraZeneca, outside the submitted work. TR reports grants and personal fees from Biosense Webster, grants from Boston Scientific, grants from Biotronik, grants and personal fees from Medtronic, grants from Abbott/SJM, personal fees from Daiichi, personal fees from BMS-Pfizer, outside the submitted work. JSt reports personal fees from Amgen, personal fees from Astra Zeneca, grants and personal fees from Bayer Healthcare, personal fees from Boehringer-Ingelheim, grants and personal fees from Biosense Webster, grants and personal fees from Boston Scientifc, personal fees from Bristol-Myers Squibb, grants and personal fees from Daiichi-Sankyo, grants and personal fees from Medtronic, personal fees from Novartis, personal fees from Pfizer, personal fees from Sanofi-Aventis, grants and personal fees from St. Jude Medical/Abbott, personal fees from Zoll, other from CorXL (ownership), grants and personal fees from Biotronik, personal fees from Atricure, personal fees from Medscape, personal fees from WebMD, personal fees from Merck/MSD, outside the submitted work. OB reports to have been an employee of CyberHeart Inc. (Sunnyvale, CA, USA) from 2008 to 2010, though he reports no financial ties or obligations or conflict of interest to or with the company or its legal predecessor Varian Inc. (Sunnyvale, CA, USA). CH-S reports personal fees from Biosense Webster, outside the submitted work. AS received educational grants from Abbott, Bayer Healthcare, Biosense Webster, Biotronik, Boston Scientific, BMS/Pfizer, and Medtronic. He owns shares from Gilead Sciences. The remaining authors declare that the research was conducted in the absence of any commercial or financial relationships that could be construed as a potential conflict of interest.
